# Natural history of treated and untreated renal oncocytoma: a systematic review and meta‐analysis

**DOI:** 10.1111/bju.16832

**Published:** 2025-07-07

**Authors:** Francesco Edwards, Jack B. Fanshawe, Joana Neves, Michael Aitchison, Soha El‐Sheikh, Archie Hughes‐Hallett, Ahmed Marous, Faiz Mumtaz, John Withington, Prasad Patki, Ravi Barod, Pedro Silva, Rebecca Varley, Wilson To, Axel Bex, Hannah Warren, Maxine G.B. Tran

**Affiliations:** ^1^ Faculty of Life Sciences and Medicine King's College London London UK; ^2^ Guy's and St Thomas’ NHS Foundation Trust London UK; ^3^ Division of Surgery and Interventional Sciences University College London London UK; ^4^ Department of Urology, Specialist Centre for Kidney Cancer Royal Free Hospital London UK; ^5^ Department of Pathology, Specialist Centre for Kidney Cancer Royal Free Hospital London UK; ^6^ Department of Urology Imperial College NHS Healthcare Trust London UK; ^7^ Department of Surgery and Cancer Imperial College London London UK; ^8^ Department of Urology University College Hospital London London UK; ^9^ Department of Urology Bart's Health NHS Trust London UK; ^10^ Bristol Urological Institute, Southmead Hospital North Bristol NHS Trust Bristol UK; ^11^ Manchester Centre for Transplantation Manchester Royal Infirmary Manchester UK; ^12^ Department of Urology Buckinghamshire Health NHS Trust Buckinghamshire UK

**Keywords:** renal, kidney, oncocytoma, oncocytic, prognosis, surveillance, ablation, surgery

## Abstract

**Introduction:**

Current guidelines recommend active surveillance, surgery, and ablation all as acceptable management strategies for renal oncocytoma, but there is growing concern about overtreatment. Our aim was to report the natural history of treated and untreated renal oncocytoma to inform clinical guidelines and shared decision‐making.

**Methods:**

A systematic review was conducted according to the Preferred Reporting Items for Systematic Reviews and Meta‐analyses (PRISMA). We systematically reviewed MEDLINE, EMBASE, CENTRAL and Clinicaltrials.gov from inception to 18 August 2023. Studies that reported outcomes during follow‐up for adult patients with treated and untreated histologically confirmed renal oncocytoma were included. The Joanna Briggs Institute tool was used to assess risk of bias for included studies. We present a narrative review and meta‐analysis.

**Results:**

There are no reports of distant metastases or disease‐related death for oncocytoma on active surveillance. Most oncocytomas on surveillance show limited growth (<2 mm/year) and minimal renal function decline (−1 mL/min/1.73m^2^/year). A significant minority (14%) transition to active treatment, most often for tumour growth. Concordance between biopsy and surgical pathology was high (89%). Metastatic oncocytoma and disease‐related death after treatment was negligible, and exclusively in reports using historic diagnostic criteria defined prior to the World Health Organisation 1998 classification, and therefore likely including eosinophilic renal cell carcinomas.

**Conclusion:**

Active surveillance of oncocytoma is oncologically safe and allows patients to avoid the risk of morbidity and mortality with treatment. Imaging surveillance after active treatment can be safely omitted. The literature would benefit from prospective cohort studies of oncocytomas on surveillance, reporting surveillance protocols, and clinical outcomes including reasons for transition to active treatment.

**Abbreviations**
AS: active surveillance
EAU: European Association of Urology
eGFR: estimated GFR
IQR: interquartile range
JBI: Joanna Briggs Institute (checklist)
PRISMA: Preferred Reporting Items for Systematic Reviews and Meta‐analyses
PROSPERO: Prospective Register of Systematic Reviews

## Introduction

Surgical resection has been the mainstay treatment for localised renal tumours for decades. However, not all renal tumours harbour malignancy, and surgical series show up to 30% are benign on surgical histopathology [1]. Renal oncocytoma is the most common type of benign renal tumour. In recent years, there has been a growing interest in preoperative detection of renal oncocytoma to allow patients to avoid the risk of morbidity associated with invasive surgical treatment [2]. However, a recent survey has demonstrated significant variation in contemporary diagnosis and management of renal oncocytoma [3].

In 2022, a systematic review and pooled analysis reported no metastases or death from biopsy confirmed renal oncocytomas on active surveillance (AS) from 10 studies with a median follow‐up of 35 months, suggesting AS is a safe and effective management option, at least the short to medium term [4]. However, there remains concern amongst the urological community about accuracy of preoperative biopsy in this setting [5], and managing biopsy confirmed oncocytoma conservatively particularly in the long term [6, 7, 8].

In the absence of evidence from randomised comparative studies, the European Association of Urology (EAU) guidelines state that AS, surgery, and ablation are all acceptable management strategies for patients with renal oncocytoma (weak recommendation) [9]. There are no guidelines on recommended follow‐up after active treatment for renal oncocytoma.

The aim of this systematic review was to report the natural history of treated and untreated oncocytoma from observational studies to inform clinical guidelines and shared decision‐making. For patients managed with active treatment, our objective was to report local and distant recurrence rates to inform the frequency and duration of post‐treatment surveillance. For patients managed with AS, our objectives were to report tumour growth over time, change in renal function, transition from AS to active treatment, and oncological outcomes for patients whilst on AS, in order to inform clinical decision making for AS. We anticipated reports of death due to oncocytoma to be rare, and in order to capture cases an additional objective was to report cases of death due to oncocytoma in autopsy studies.

## Methods

### Protocol and Registration

The protocol was developed according to Preferred Reporting Items for Systematic Reviews and Meta‐analyses (PRISMA) guidelines [10] and prospectively registered with the International Prospective Register of Systematic Reviews (PROSPERO; CRD42017058723).

### Search Strategy and Sources

Comprehensive searches of electronic databases MEDLINE and EMBASE, The Cochrane Register (CENTRAL), and Clinicaltrials.gov were performed from inception to 18 August 2023. Where necessary, the authors of potentially useful studies were contacted for information. Search strategies are detailed in the Fig. S1 and Table S1. Controlled and uncontrolled vocabulary were used. Search terms were limited to humans and the English language.

Returned articles from each database were combined and duplicates removed using systematic review management software Rayyan [11].

### Eligibility Criteria

All primary research articles including adult patients (aged ≥18 years) with a histologically confirmed diagnosis of renal oncocytoma through biopsy or surgical pathology, managed with AS or active treatment with any period of clinical follow‐up beyond an initial post‐surgical follow‐up visit were included. Studies reporting only immediate peri‐procedural outcomes without long‐term follow‐up were excluded. Any definition of renal oncocytoma was included; however, reports that explicitly included hybrid oncocytic‐chromophobe RCC tumours, oncocytic RCC, or oncocytomas only in the context of the Birt–Hogg–Dubé syndrome were excluded. Studies were not restricted in terms of publication status, setting, design, or quality. Studies describing growth rates, progression (according to TNM staging), management, and follow‐up of renal oncocytoma were included. Case reports, editorials, letters to the editor, and review articles were excluded. Full manuscripts and conference abstracts with sufficient information to meet the inclusion criteria were included.

### Study Selection

Titles and abstracts were screened by two independent members of the research team, with conflicts resolved by a third author. Potentially relevant full texts were then screened in the same manner. Duplicates were removed. Where multiple publications from the same authors and institution were found with an overlapping recruitment period, the report with the smaller sample size was excluded due to likely overlapping cases. Reasons for exclusions were recorded. Hand searches of reference lists of included studies were performed to identify additional relevant literature.

### Data Collection

Data were extracted using a pre‐piloted form by two independent authors, disagreements were discussed with a third reviewer to reach consensus. Further information was requested from the corresponding authors of the study if required.

The following data were extracted: study characteristics (author, publication year, journal, institution, single‐ or multicentre, country where research was conducted, language of publication, study period, study design, sample size), patient characteristics (age, gender, tumour size, multifocality, presenting symptoms, family history, personal history of RCC, estimated GFR [eGFR measured in mL/min/1.73 m^2^]), chosen initial management (AS, ablation, surgery, or if the oncocytoma was diagnosed at autopsy), the means of oncocytoma diagnosis (biopsy, surgical specimen) was recorded, and the diagnostic criteria used. The follow‐up strategy was also extracted, including mode of imaging (ultrasonography, CT, MRI) and imaging frequency. Patient outcomes were recorded including the proportion of included patients who died from any cause, died due to oncocytoma, and developed local recurrence/progression, or distant metastases during follow‐up. For patients with a period of AS, the proportion of oncocytomas demonstrating interval growth, growth rate, change in eGFR, conversion to active treatment, and reasons thereof were collected.

### Risk of Bias

Risk of bias was assessed by two independent members of the research team, using the Joanna Briggs Institute (JBI) checklist for critical appraisal of case series [12]. A third author acted as adjudicator in the case of disagreement. The results were visualised using the ‘robviz’ tool [13].

### Data Synthesis

Measures of interest included population baseline characteristics, duration of follow‐up (months), annual growth rate (mm/year), annual change in eGFR (mL/min/1.73m^2^/year), and proportion of patients developing oncological outcomes (metastases and disease‐specific death).

For analysis of proportions of binomial data, the ‘metaprop’ command in Stata version 18.0 (Stata Corp., College Station, TX, USA) was used. Random effects models were used due to between‐study heterogeneity. The Freeman–Tukey double arcsine transformation was applied when a study reported a proportion close to 0 or 1, and CIs calculated using the Score method [14]. For meta‐analysis of study population averages (reported as median or mean) for near‐normally distributed data, median and interquartile ranges (IQRs) were approximated to means and SDs using methods described by Wan et al. [15] and the ‘metan’ package in Stata version 18 was used. This method was used for all studies, including those in which the study authors reported summary estimates and 95% CIs computed by alternative methods. For non‐normally distributed data, we present the median and IQR of study reported averages.

Heterogeneity was assessed by visual inspection of the results and calculation of the *I* ^2^ statistic. Reporting is according to the PRISMA guidelines.

## Results

### Study Selection

**Fig. 1:**
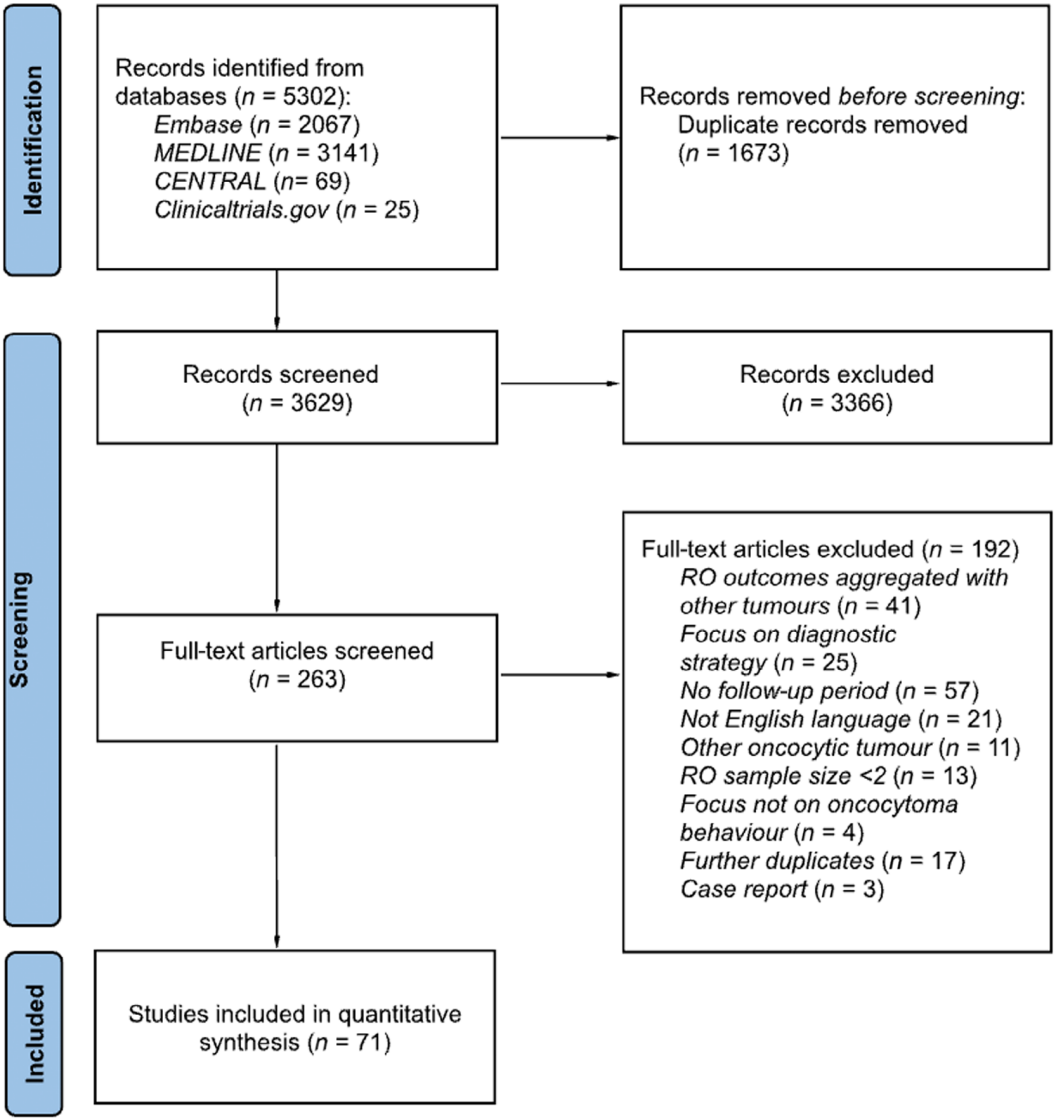
The PRISMA flow diagram describing study selection process and reasons for exclusion.

The search identified 5302 potential studies. Following removal of duplicates, 3366 studies underwent title and abstract screening, followed by 191 full texts. A total of 71 studies were included, including 3812 renal oncocytomas in 3310 individuals. Reasons for exclusion of studies from the review are shown in Fig. 1.

### Study Characteristics

Participant demographics in included studies were as follows: 62% male (95% CI 59–65%), mean age at diagnosis 67 (95% CI 64–69) years, and mean tumour size at diagnosis 49 (95% CI 39–58) mm. Multifocal oncocytomas were present in 9% (95% CI 2–17%), and bilateral oncocytoma in 7% (95% CI 2–15%). Patients were followed up for a mean of 43 months (IQR 37–62 months). All studies were retrospective observational case series or cohort studies. Individual study characteristics are reported in Table S1.

### Untreated Renal Oncocytoma

Patient outcomes on AS were described for 932 oncocytomas in 23 studies with a median (IQR) follow‐up of 35 (30–40) months [6, 16, 17, 18, 19, 20, 21, 22, 23, 24, 25, 26, 27, 28, 29, 30, 31, 32, 33, 34, 35, 36, 37]. There were no reported metastases or deaths due to oncocytoma on AS during follow‐up.

**Fig. 2:**
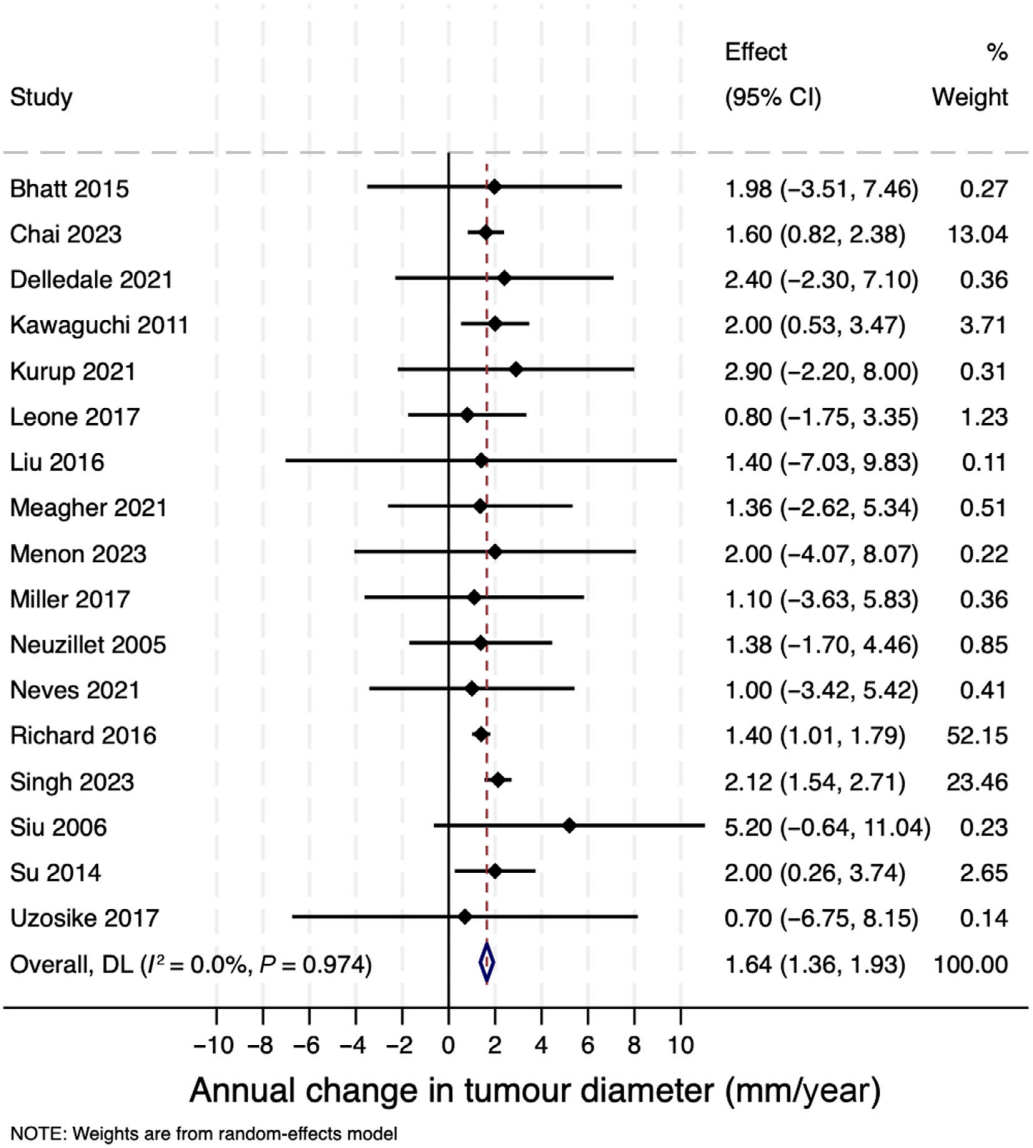
Estimates of mean annual change in tumour diameter (mm/year) for oncocytomas on AS.

The mean growth rate on AS, defined as the mean change in the maximum tumour diameter per year, was reported for 725 oncocytomas in 17 studies at 1.6 mm/year (95% CI 1.4–1.9 mm/year; Fig. 2) [16, 17, 18, 20, 21, 22, 23, 24, 25, 27, 28, 29, 34, 35, 36, 37, 38].

The mean change in eGFR per year during follow‐up was reported for 186 oncocytomas on surveillance in four studies using the same units of measurement, with an average of −0.99 mL/min/1.73 m^2^/year (95% CI −2.21 to +0.23 mL/min/1.73 m^2^/year) [19, 22, 23, 26]. For comparison, healthy population‐level data reports mean eGFR change over time of −1 to −1.2 mL/min/1.73m^2^/year, increasing with increasing age [39, 40].

During follow‐up 14% of patients (95% CI 5–26%) on AS for a biopsy diagnosed oncocytoma converted to active treatment (16% partial nephrectomy, 11% radical nephrectomy, 26% ablation, 40% unspecified surgery, 10% unreported) [6, 16, 17, 18, 19, 21, 23, 25, 27, 29, 38]. The most common described reason for conversion to active treatment was tumour growth in 64%, although the absolute growth in these cases was infrequently defined. Other reported reasons for transition to active treatment included patient preference in 6%, presence of symptoms in 6%, change in radiological appearance in 4%, large tumour size at diagnosis in 2%, independent review of biopsy pathology suggesting raised possibility of chromophobe RCC in 1%, and unreported or unclear in 16%. The median (IQR) time to active treatment was 27 (23–28) months for the nine studies including 117 oncocytomas that reported the time to transition from AS to active treatment.

### Treated Renal Oncocytoma

#### Ablation

Thermal ablation was used to treat 144 oncocytomas in 16 studies; 50% radiofrequency (95% CI 5–96%), 20% cryotherapy (95% CI 0–55%), and otherwise the energy source was not clearly reported [6, 16, 17, 18, 20, 21, 24, 27, 28, 30, 34, 35, 38, 41, 42, 43]. The approach was described as percutaneous in 73% (95% CI 12–100%), laparoscopic in 11% (95% CI 0–63%), surgical in 6% (95% CI 0–45%), and not reported in 20% (95% CI 0–63%).

The mean post‐treatment follow‐up was 45 months (IQR 28–46.5 months). Major peri‐procedural complications were reported after ablation of renal oncocytoma in 3% (95% CI 0–18%); including a duodenal fistula requiring drainage and interval nephrectomy [26], and one postoperative death relating to complications from general anaesthesia [19]. For oncocytomas treated with ablation, local or distant recurrence was reported in 0% (95% CI 0–0%), and there was no disease‐specific mortality reported during follow‐up.

#### Surgery

Surgical management was reported for 2658 oncocytomas in 60 studies [6, 16, 17, 18, 19, 20, 21, 22, 24, 25, 27, 28, 30, 31, 32, 34, 35, 38, 44, 45, 46, 47, 48, 49, 50, 51, 52, 53, 54, 55, 56, 57, 58, 59, 60, 61, 62, 63, 64, 65, 66, 67, 68, 69, 70, 71, 72, 73, 74, 75, 76, 77, 78, 79, 80, 81, 82, 83, 84, 85]; partial nephrectomy in 29% (95% CI 16–43%), radical nephrectomy in 70% (56–83%). Major postoperative complications (Clavien–Dindo Grade ≥III) were reported in 4% (95% CI 1–8%) [22, 24, 55, 75, 80], including perioperative deaths 0% (95% CI 0–3%) [45, 51, 66].

**Fig. 3:**
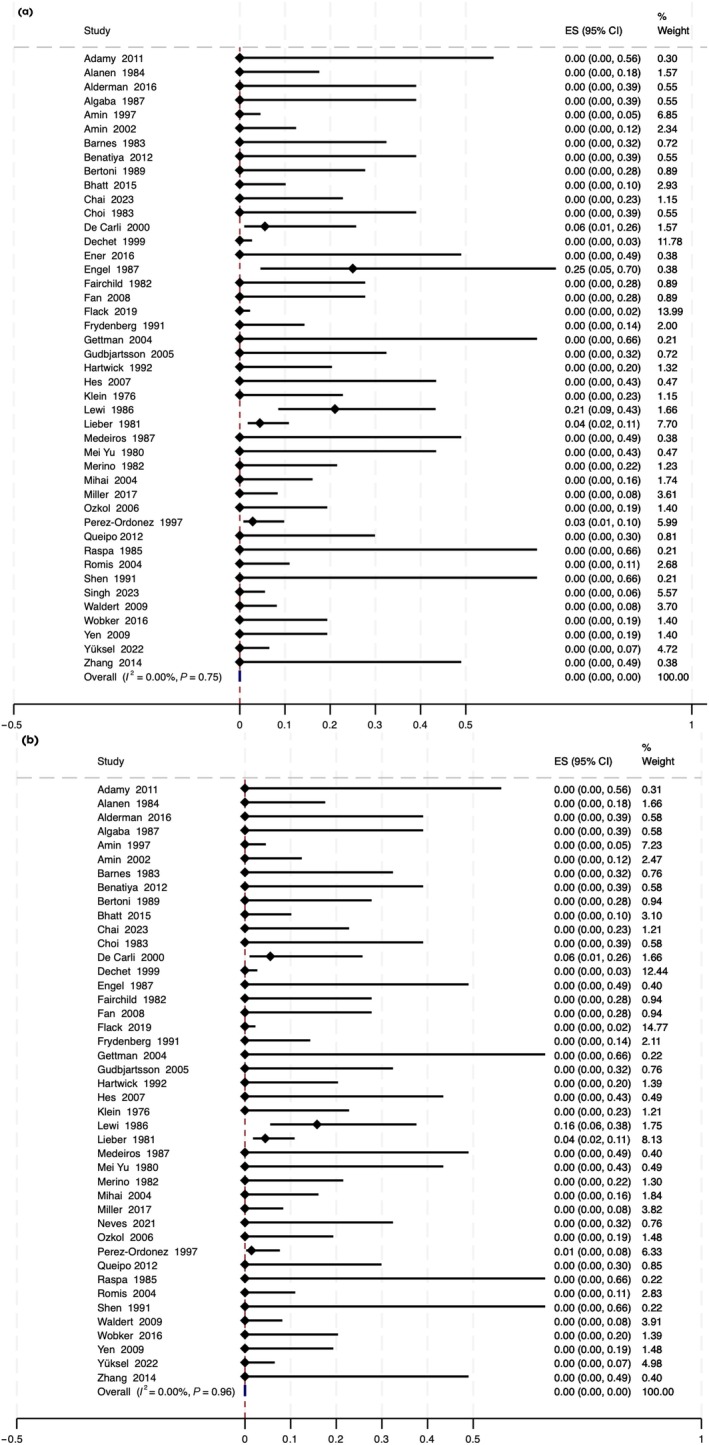
Proportion of patients with surgically treated renal oncocytoma reported to have (**a**) distant metastases and (**b**) disease‐specific death. Note that studies reporting distant metastases and death from renal oncocytoma were exclusively diagnosed prior to the introduction of the WHO classification system 1998 when oncocytoma, chromophobe RCC, and granular cell RCC were considered distinct entities for the first time [86].

During a median (IQR) postoperative follow‐up of 50 (33–80) months, local recurrence was reported in no case (0% [95% CI 0–0%]), distant recurrence in 12 cases (0%, 95% CI 0–0%) [54, 58, 66, 67, 73] (Fig. 3a), and disease‐specific mortality in nine (0%, 95% CI 0–0%) [54, 66, 67, 73] (Fig. 3b). Distant recurrence and metastasis were reported exclusively in studies using diagnostic criteria prior to the WHO classification system of 1998, when oncocytoma, chromophobe RCC, and granular cell RCC were considered distinct entities for the first time [86].

There were three reports that focused on asynchronous renal tumours in 5% (95% CI 4–8%) of patients with a history of surgically treated oncocytoma (20 oncocytomas, six RCCs, 15 without histological diagnosis), during an average follow‐up of 57 months [52, 55, 77]. For comparison the population‐level lifetime risk of developing a kidney tumour is ~2% in the absence of population‐level screening [87].

For studies that reported surgical pathology diagnosis for biopsy diagnosed oncocytoma, the pooled estimate of concordance was 89% (95% CI 80–96%) [6, 17, 18, 19, 20, 21, 24, 25, 26, 27, 30, 31, 35, 46, 61]. Of the 31 discordant cases, 28 were oncocytic tumours (eight hybrid oncocytic‐chromophobe RCC, 16 chromophobe RCC, one low‐grade oncocytic tumour, two oncocytic papillary RCC, one oncocytoma with metastatic lobular breast carcinoma), two clear cell RCCs, and one unclassified tumour.

### Diagnosis at Autopsy

Nine studies reported data for 40 renal oncocytomas diagnosed at autopsy [45, 49, 53, 56, 58, 61, 63, 64, 66]. All were localised tumours, and the cause of death was not related to oncocytoma in any case.

### Risk of Bias Assessment

The JBI critical appraisal checklist for case series revealed a range of quality in the assessment of bias. The tool assesses 10 domains for each study that are markers of quality that can be classified as either ‘met’, ‘not met’, or ‘unclear’. Studies are assigned a percentage score indicating the number of criteria met, with lower scores indicating a higher risk of bias. Included studies met 30–100% of criteria (median 80%). In general, the condition was measured using standard and valid methods, i.e., histopathology reporting of biopsy/surgical specimens using accepted diagnostic criteria. Inclusion criteria, patient demographics, clinical information, and outcomes were clearly reported. Presenting site/clinic demographic information was poorly reported, and in many cases no statistical analysis was performed. All included reviews were retrospective, with an increased risk of bias due to potential missing data, alongside selection, information, and confounding bias. A summary of critical appraisal results is shown in Fig. S1.

## Discussion

### Main Findings

There are no reports in the published literature of metastasis or disease‐related death for renal oncocytomas managed with AS. There are no reported deaths due to renal oncocytoma in autopsy studies. Oncocytomas on AS exhibit minimal growth (average <2 mm/year). A significant minority transition from AS to active treatment (14%) in the medium term, with the most commonly reported reason of tumour growth likely reflecting uncertainty about the natural history of these benign tumours in the longer term. Concordance between surgical pathology and biopsy diagnosis of renal oncocytoma was high and in cases of discordance, the majority were good‐prognosis oncocytic spectrum tumours.

There are no reports in the published literature describing local recurrence after active treatment. Distant recurrence and disease‐specific death after surgery for oncocytoma was reported in <1% of cases, exclusively in reports using diagnostic criteria pre‐dating the 1998 WHO classification system and were therefore likely misclassified tumours [86].

### General Interpretation in the Context of Other Evidence

This is the first systematic review to report the natural history of treated and untreated renal oncocytoma.

Our findings relating to untreated oncocytoma are consistent with a previous systematic review and pooled analysis published in 2022, which included 633 oncocytomas that concluded AS of renal oncocytoma is safe in the medium term [4]. All reports included in the previous review were included in our study, in addition to new evidence [6, 17, 19, 23, 28, 31, 33, 47] and reports that were not included in the previous systematic review; one study published in abstract form only [29], one study in which just one oncocytoma was managed with AS [32], and three studies seemingly missed [16, 36, 37]. We therefore present the most recent and comprehensive review of outcomes for patients managed with AS for renal oncocytoma. Our results further support the of safety of managing oncocytomas with AS.

Managing localised renal oncocytoma with AS should also be viewed within the context of a growing appetite to manage more localised renal masses conservatively with AS, regardless of tumour subtype [88]. Ageing populations with increasing comorbidity profiles means that competing cause mortality often predominates over risk of death from a localised renal tumour [89], which is particularly pertinent if the tumour subtype is indolent or benign.

There have been no previous systematic reviews reporting long‐term patient outcomes after active treatment for oncocytoma. Reflecting this evidence gap, follow‐up regimens after active treatment vary, from discharge after the first post‐treatment review to regular surveillance for up to 5 years [3]. This is particularly pertinent in cases where there is histological evidence of fat and vascular invasion. Our review found no reports of local recurrence, or distant metastases, or disease‐specific death in studies using modern diagnostic criteria. Therefore, our review would support omission of imaging surveillance in patients who have completed active treatment for renal oncocytoma, particularly when diagnosed according to contemporary standards, although clinicians will be mindful of the small risk of misdiagnosis of biopsy confirmed oncocytomas treated with ablation.

### Limitations of Review Processes and the Evidence Included in the Review

The decision to include older studies in the review was made with the objective of reporting long‐term follow‐up data. However, this has meant the evidence presented in this review is affected by diagnostic drift in the criteria used to classify renal oncocytoma, with the WHO classification system for urinary and male genital tumours being updated in 1998, 2004, 2016, and 2022 after its original publication in 1981 [90]. Early editions of the WHO classification system relied solely on morphological diagnostic criteria described on light microscopy and did not include immunohistochemistry, which is now considered a contemporary standard. As a result, older reports included in this review likely misclassified eosinophilic variants of RCC as oncocytoma, as they have similar morphological features [91]. In this review, we did not identify any reports of distant metastases or disease‐specific death for oncocytomas diagnosed after 1998.

Despite advances in immunohistochemistry and molecular diagnoses, the latest WHO classification system published in 2022 cautions against making a definite diagnosis of oncocytoma on core biopsy specimens due to intratumoral heterogeneity of eosinophilic variants of RCC, which can have areas of similar appearance to oncocytoma [92]. This caution is echoed in 2024 EAU guidelines [9], presenting a challenge for contemporary practice and frustrating efforts to reduce overtreatment of benign oncocytoma [7]. However, a more pragmatic approach has recently been suggested whereby all oncocytic‐spectrum tumours diagnosed on biopsy represent a single entity of tumours with low malignant potential, best managed with AS [93]. By including older studies in our review, that grouped benign oncocytomas and misclassified malignant eosinophilic RCCs together as a single entity, we demonstrate that even when malignant eosinophilic tumour subtypes are misclassified as oncocytoma, the number of metastatic events and disease‐related mortality was negligible. Therefore, whilst definite diagnosis of low‐grade oncocytic tumour subtypes may be of academic interest, the evidence presented in this review suggest it is rarely clinically significant [94].

We did not separately assess outcomes for patients on AS following a biopsy diagnosis of oncocytoma vs those who had a period of AS for a histology agnostic tumour prior to surgical histopathology. There is likely selection bias in patients and tumours selected for up‐front biopsy and AS prior to surgery. Similarly there is a selection bias in patients who underwent surgery after a period of AS for biopsy diagnosed oncocytoma.

There are no randomised comparative trials for active treatments vs AS for renal oncocytoma. Included reports were retrospective inception cohort studies and case series that are limited by selection bias, missing data, loss to follow‐up, and heterogeneity between studies.

The focus of our review was to report long‐term outcomes for patients diagnosed with renal oncocytoma. We therefore excluded studies reporting only short‐term measures of morbidity and mortality following active treatment for oncocytoma without longer‐term follow‐up for oncological outcomes, therefore our estimates of perioperative complication risk are not a comprehensive summary of the published literature.

A population‐based study in the UK reported outcomes following surgery for 1202 renal oncocytoma over the 4 years to 2016, which reported a 4% risk of major complications (Clavien–Dindo Grade ≥III) and a 0.3% risk of 30‐day mortality following surgery [2]. These findings were comparable to outcomes following all renal surgery reported in the same national registry [95, 96]. Whilst peri‐procedural complications following ablation procedures for renal oncocytoma specifically have not been reported, a systematic review and meta‐analysis of outcomes following management for any localised renal tumour reported risk of major postoperative complications (Clavien–Dindo Grade III–IV) following partial nephrectomy, radical nephrectomy, and thermal ablation of 1.1–6.9%, 2.8–5.1%, and 1.3–11.1% [97], respectively, which is consistent with the findings in our review.

Four studies, including 186 patients with oncocytoma, reported change in eGFR whilst on AS, with the pooled estimate suggesting that renal function decline is the same for patients with untreated oncocytoma as in the general population [39, 40]. This finding supports the safety of AS for oncocytoma in terms of renal function.

The evidence included in our systematic review is limited to retrospective cohorts and case series (Level of evidence 2 and 4, respectively) (169), limiting the strength of any recommendation that can be made [98].

### Implications of the Results for Practice, Policy, and Future Research

This systematic review has shown that AS for renal oncocytoma is safe whilst allowing patients to avoid the risk of morbidity and mortality from active treatment. Therefore, the review supports a change in clinical guidelines to recommend AS as first‐line treatment for newly diagnosed, asymptomatic oncocytoma.

We would recommend further research in this field including prospective registries reporting growth dynamics, clear reasons for transition to active treatment, renal function dynamics (reported as mL/min/1.73m^2^ per year to facilitate future systematic reviews), and frequency and modality of imaging over the long term to improve the quality of the current evidence base and strength of guideline recommendations. Such work would also help inform additional guidelines on follow‐up regimen and triggers to intervene that at present are uncertain and at the discretion of the managing clinician and local practices. Future work should also report AS outcomes for patients diagnosed with oncocytic tumours on biopsy according to the WHO 2022 classification system, and emerging diagnostic tools such as 99mTc‐sestamibi single‐photon emission CT (SPECT)/CT and ‘virtual biopsy’ [99].

## Conclusion

Our data suggests that AS of oncocytoma is safe and avoids the risk of major morbidity and mortality, albeit small, from active treatment.

Whether it is possible to make a definite diagnosis of oncocytoma from other low‐grade eosinophilic tumours on core biopsy appears to be of limited clinical relevance, as our review included reports from study periods when these tumours were considered a single entity.

Furthermore, in cases of treated oncocytoma, evidence in this review supports discharge following recovery from treatment with surgery, negating the need for long‐term follow‐up. Discharge following ablation for biopsy diagnosed oncocytomas is also likely to be safe but caveated by a small risk of tumour misclassification with biopsy.

## Disclosure of Interests

Jack B. Fanshawe is supported by a UK National Institute for Health and Care Research (NIHR) Academic Clinical Fellowship (ACF‐2023‐13‐016). Hannah Warren is supported by research fellowships from The Urology Foundation, The Pan London Cancer Alliance, Wellcome/Engineering and Physical Sciences Research Council (EPSRC) Centre for Interventional and Surgical Sciences and Royal College of Surgeons of England. Maxine G.B. Tran is a panel member on the European Urology Kidney Cancer Guidelines and the National Institute for Health and Care Excellence (NICE) Kidney Cancer Guideline Committee. Maxine G.B. Tran has received honorarium for educational engagements and events from MSD and Boston Scientific. The other authors have no conflicts of interest to declare.

## Supporting information


**Fig. S1.** Risk of bias assessment for included studies using the Joanna Brigg's Institute critical appraisal checklist for case series.

**Table S1.** Characteristics of included studies.
