# Supplementary material for: Natural history of treated and untreated renal oncocytoma: a systematic review and meta‐analysis
Source: BJU Int. 2025 Jul 7;136(4):590–601. doi: 10.1111/bju.16832 (PMC12415324; doi:10.1111/bju.16832)
Supplement: Supplementary file 1 — Fig. S1. Risk of bias assessment for included studies using the Joanna Brigg's Institute critical appraisal checklist for case series. [file BJU-136-590-s001.docx]

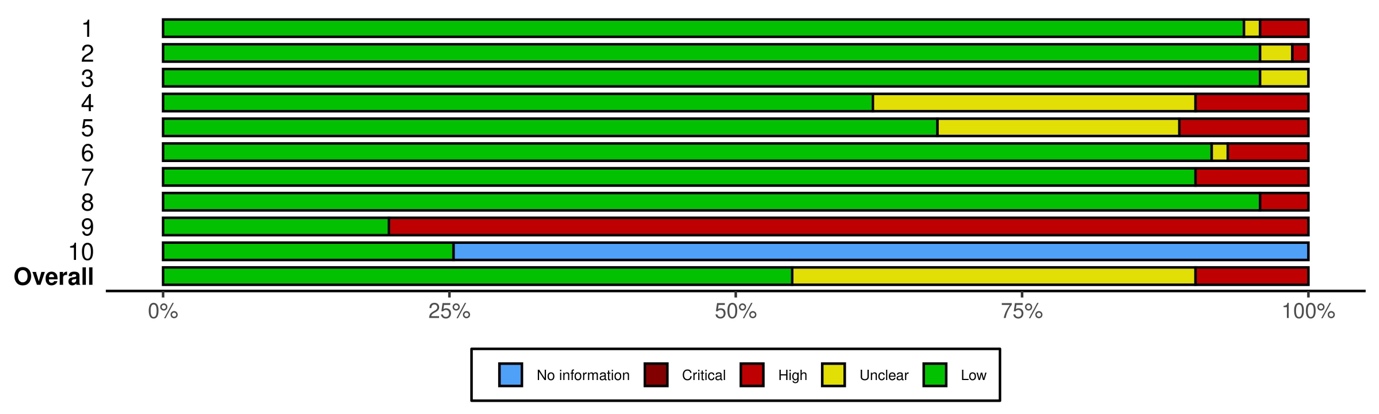


Figure S 1: Risk of bias assessment for included studies using the Joanna Brigg’s Institute critical appraisal checklist for case series. Items 1-10 as follows:

1) Were there clear criteria for inclusion?

2) Was the condition measured in a standard, reliable way for all participants?

3) Were valid methods used for identification of the condition for all participants?

4) Was there consecutive inclusion of participants?

5) Was there complete inclusion of participants?

6) Was there clear reporting of the demographics of the participants in the study?

7) Was there clear reporting of clinical information of the participants?

8) Were the outcomes or follow up results of cases clearly reported?

9) Was there clear reporting of the presenting site(s)/clinic(s) demographic information?

10) Was statistical analysis appropriate?
