# Supplementary material for: Natural history of treated and untreated renal oncocytoma: a systematic review and meta‐analysis
Source: BJU Int. 2025 Jul 7;136(4):590–601. doi: 10.1111/bju.16832 (PMC12415324; doi:10.1111/bju.16832)
Supplement: Supplementary file 2 — Table S1. Characteristics of included studies. [file BJU-136-590-s002.docx]

Supplementary Table 1: Characteristics of included studies. Note sum of the number of tumours managed with surveillance, ablation and surgery may not add to the sample size as a) some patients had multiple tumours b) some cases were lost to follow up c) some patients embarked on a period of active surveillance, later transitioning to active treatment in which case they are ‘double counted’ d) some studies aggregated management data for oncocytomas with tumours of other histotypes so it was not possible to report management choice for the oncocytomas alone.

RN = radical nephrectomy, PN = partial nephrectomy, RFA = radiofrequency ablation, LCA = laparoscopic cryoablation, PCA = percutaneous cryoablation IQR = interquartile range SD = standard deviation

| **Author and year of publication** | **Study country and dates** | **Sample Size** (number of included oncocytomas) | **Average age (years)** | **% male** | **Average tumour diameter at baseline (range, unless specified), mm** | **Number managed with Active Surveillance** | **Number managed with Ablation** | **Surgically Treated** | **Average follow up period (months)** | **Diagnosed at autopsy** |
| --- | --- | --- | --- | --- | --- | --- | --- | --- | --- | --- |
| Abel et al. 2017 (101) | USA 2003-2016 | 125 | NR | NR | NR | NR | NR | NR | 48 | 0 |
| Adamy et al. 2011 (44) | USA 1995-2009 | 3 | NR | NR | NR | 0 | 0 | 3 | NR | 0 |
| Alanen et al. 1984 (45) | Finland 1966-1982 | 30 | NR | 43 | NR (5-250) | 0 | 0 | 18 | NR | 12 |
| Alderman et al. 2016 (30) | USA 2006-2013 | 96 | NR | NR | NR | 58 | 20 | 6 | 36 | 0 |
| Algaba et al. 1987 (46) | Spain 1978-1985 | 7 | 64 | 29 | 75 (55-125) | 0 | 0 | 7 (7 RN) | 22 | 0 |
| Amin et al. 1997 (48) | USA 1975-1990 | 80 | 67 | 75 | 44 (6-150) | 0 | 0 | 80 (68 RN, 12 PN) | 91 | 0 |
| Amin et al. 2002(102) | USA 1968-1994 | 27 | 66 | 70 | 49 (20-200) | 0 | 0 | 27 | 120 | 0 |
| Barnes et al. 1983 (49) | USA 1947-1981 | 12 | 58 | 100 | 65 (30 – 110) | 0 | 0 | 8 | 24 | 4 |
| Benatiya et al. 2012 (50) | Morocco 1990-2008 | 6 | 53 | NR | 88 (65-120) | 0 | 0 | 6 (6 RN) | 36 | 0 |
| Bertoni et al. 1989 (51) | Italy 1975-1986 | 10 | 60 | 50 | 100 (40-200) | 0 | 0 | 10 (10 RN) | 49 | 0 |
| Bhatt et al. 2015 (16) | Ireland 1998-2015 | 38 | 57 | 50 | 47 (15-180) | 4 | 1 (RFA) | 34 (27 RN, 7 PN) | 84 | 0 |
| Bosniak et al. 1995 (103) | USA 1981-1993 | 4 | 61 | NR | 27 (16-40) | 0 | 0 | 4 | NR | 0 |
| Branger et al. 2023 (6) | France | 119 | 64 | 54 | 40 (NR) | 65 | 6 | 75 (4 RN, 71 PN) | 20 | 0 |
| Chai et al. 2023 (17) | USA Dates NR | 107 | NR | NR | 24 (IQR 10 – 109) | 68 | 16 | 13 | NR | 0 |
| Childs et al. 2010(52) | USA 1970-2007 | 424 | 68 | 68 | 35 (4 – 160) | 0 | 0 | 424 | 85 | 0 |
| Choi et al. 1983 (53) | USA 1965-1981 | 7 | 69 | 100 | 30 (3 – 200) | 0 | 0 | 5 | 60 | 2 |
| Daza et al. 2022 (33) | USA 2013-2021 | 43 | NR | NR | NR | 43 | 0 | 0 | NR | 0 |
| De Carli et al. 2000 (54) | Italy 1993-1999 | 18 | NR | 50 | 40 (15-120) | 0 | 0 | 18 (12 RN, 6 PN) | 46 | 0 |
| Dechet et al. 1999 (55) | USA 1980-1997 | 138 | 68 | 72 | 32 (60-145) | 0 | 0 | 138 (83 RN, 55 PN) | 41 | 0 |
| Deledalle et al. 2021 (18) | France 2010-2016 | 89 | 67 | 57 | 26 (15-90) | 89 | 11 (2 PCA, 9 RFA) | 13 (5 RN, 8 PN) | 36 | 0 |
| Ejeckam et al. 1979 (56) | Canada Dates NR | 8 | 65 | 63 | NR (25-100) | 0 | 5 | 0 | NR | 3 |
| Ener et al. 2016 (57) | Turkey 2009-2015 | 4 | NR | NR | NR | 0 | 0 | 4 (4 PN) | NR | 0 |
| Engel et al. 1987 (58) | Denmark Dates NR | 5 | 75 | 40 | 51 (20-100) | 0 | 0 | 4 | 39 | 1 |
| Fairchild et al. 1983 (59) | USA 1952-1984 | 13 | NR | 73 | NR | 0 | 0 | 12 (11 RN, 1 PN) | NR | 0 |
| Fan et al. 2008 (60) | Taipei 1988-2006 | 13 | 60 | 77 | 53 (27-85) | 0 | 0 | 13 | 53 | 0 |
| Fernando et al.2012 (41) | Not reported | 3 | NR | NR | NR | 0 | 3 (LCA) | 0 | NR | 0 |
| Flack et al. 2019 (84) | USA 2000-2016 | 164 | 65 | 66 | 35 (SD 23) | 0 | 0 | 164 (50 RN, 114 PN) | 33 | 0 |
| Frydenberg et al. 1991 (61) | Australia 1978-1984 | 24 | 65 | 58 | 48 (15-115) | 0 | 0 | 23 (19 RN, 4 PN) | 34 | 1 |
| Gettman et al. 2004 (62) | USA 2002-2003 | 2 | NR | NR | NR | 0 | 0 | 2 (2 PN) | NR | 0 |
| Ginsburg et al. 2022 (19) | USA Dates NR | 114 | NR | NR | NR | 32 | 0 | 82 (82 PN) | NR | 0 |
| Gudbjartsson et al. 2005 (63) | Iceland 1971-2000 | 45 | 74 | 69 | 51 (6-180) | 0 | 0 | 31 (30 RN, 1 PN) | 99 | 14 |
| Hartwick et al. 1992 (64) | Canada/US 1985-1989 | 23 | 64 | NR | 64 (20-120) | 0 | 0 | 21 (21 RN) | 50 (12-144) | 2 |
| Hes et al. 2007 (104) | Czech Rep/Italy/ Japan/ Mexico/US Dates NR | 7 | 73 | 71 | 49 (22-72) | 0 | 0 | 7 (7 RN) | 43 (12-60) | 0 |
| Huang et al. 2015 (105) | USA 2000-2010 | 102 | 64 | NR | NR | NR | NR | NR | 60 (0-156) | 0 |
| Kawaguchi et al. 2011 (34) | Canada 2004-2010 | 30 | 73 | 62 | 26 (6-80) | 24 | 2 (thermal) | 6 (6 RN) | 40 (12-119) | 0 |
| Klein et al. 1976 (85) | USA 1964-1976 | 14 | 63 | 64 | 70 (35-130) | 0 | 0 | 14 | 21 (0-132) | 0 |
| Kurup et al. 2012 (20) | USA 2000-2009 | 33 | 71 | NR | 17 (4 – 47) | 33 | 14 (5 RFA, 9 PCA) | 19 (5 RN, 14 PN) | 33 (12-124) | 0 |
| Leone et al. 2017 (35) | USA 2005-2013 | 32 | NR | 83 | NR | 5 | 2 (RFA) | 12 (1 RN, 11 PN) | 34 | 0 |
| Lewi et al. 1986 (66) | Scotland 1965-1984 | 22 | NR | 50 | NR | 0 | 0 | 21 (21 RN) | NR | 1 |
| Lieber et al. 1981 (67) | USA 1950-1979 | 90 | 52 | 63 | 60 (30-260) | 0 | 0 | 90 (90 RN) | NR | 0 |
| Liu et al. 2016 (21) | Australia 2000-2014 | 53 | 64 | NR | 34 (13-87) | 53 | 1 (RFA) | 5 (1 RN, 4 PN) | 34 (6-109) | 0 |
| Meagher et al. 2021 (22) | USA, Italy 2006-2018 | 295 | 67 | 64 | 29 (NR) | 71 | 0 | 224 (224 PN) | 37 | 0 |
| Medeiros et al. 1987 (68) | USA 1960-1978 | 6 | 56 | NR | 56 (15-70) | 0 | 0 | 6 (6 RN) | 175 (57-285) | 0 |
| Mei Yu et al. 1980 (69) | USA 1963-1977 | 5 | 62 | 100 | 81 (50-106) | 0 | 0 | 5 (5 RN) | 132 (24-156) | 0 |
| Menon et al. 2023 (23) | USA Dates NR | 32 | 73 | 47 | 31 (IQR 23-43) | 32 | 0 | 0 | 37 (21-47) | 0 |
| Merino et al. 1982 (70) | USA 1958-1979 | 14 | 60 | 57 | NR (24-140) | 0 | 0 | 14 (14 RN) | 60 (12-168) | 0 |
| Mihai et al. 2004(71) | Romania 2000-2004 | 20 | 66 | 40 | 61 (35-100) | 0 | 0 | 20 (20 RN) | 13 (6-35) | 0 |
| Miller et al. 2018(24) | USA 2003-2016 | 122 | 69 | 66 | NR | 81 | 24 | 42 (19 RN, 23 PN) | 39 (14-71 IQR) | 0 |
| Moon et al. 2004(42) | USA 2000-2002 | 2 | 68 | NR | 18 (15-20) | 0 | 2 (LCA) | 0 | 11 (7-15) | 0 |
| Nazzani et al. 2022 (31) | Italy 2008-2017 | 15 | NR | NR | NR | 8 | 0 | 7 (2 RN, 5 PN) | NR | 0 |
| Neuzillet et al. 2005 (25) | France 1998-2004 | 15 | 57 | 40 | 35 (12-100) | 15 | 0 | 6 (4 RN, 2 PN) | 40 (±21 SD) | 0 |
| Neves et al. 2021 (26) | UK 2012-2019 | 101 | 69 | 70 | 34 (5-88) | 98 | 1 (PCA) | 8 (5 RN, 3 PN) | 29 (2-89) | 0 |
| Ozkol et al. 2006(72) | Turkey 1991-2004 | 16 | 65 | 19 | 57 (SD 28.8) | 0 | 0 | 16 (11 RN, 5 PN) | 80 (±48 SD) | 0 |
| Perez-Ordonez et al. 1997 (73) | USA 1980-1995 | 70 | 65 | 56 | 52 (15-140) | 0 | 0 | 70 (61 RN, 9 PN) | 50 (1-181) | 0 |
| Queipo et al. 2012 (74) | Spain Dates NR | 9 | 73 | 56 | 42 (16-115) | 0 | 0 | 9 | 11 (3-108) | 0 |
| Raspa et al. 1985 (75) | USA 1971-1976 | 2 | 59 | 50 | NR | 0 | 0 | 4 (2 RN, 2 PN) | 126 (96-156) | 0 |
| Richard et al. 2016 (27) | Canada 2003-2014 | 81 | 68 | 61 | 2.4 (IQR 1.6-3.7) | 81 | 2 (RFA) | 4 | 34 (25-58 IQR) | 0 |
| Romis et al. 2004 (32) | Italy/France 1987-2001 | 32 | 60 | NR | 49 (20-140) | 1 | 0 | 31 (27 RN, 4 PN) | 42 (8-76) | 0 |
| Shen et al. 1991 (76) | China Dates NR | 2 | 55 | 0 | 88 (85-90) | 0 | 0 | 2 (2 RN) | 66 (36-96) | 0 |
| Singh et al. 2023 (28) | USA Dates NR | NR | 64 | 80 | NR | 19 | 2 | 65 (13 RN, 52 PN) | 68 | 0 |
| Siu et al. 2006 (36) | USA 1996-2005 | 6 | 69 | NR | 22 (10-45) | 6 | 0 | 0 | 31(13-60) | 0 |
| Su et al. 2014 (29) | NR 1999-2013 | 29 | 63 | 83 | 44 (18-100) | 29 | 0 | 0 | 52 (10-123) | 0 |
| Tan et al. 2012 (43) | USA 2001-2011 | 37 | NR | NR | NR | 0 | 37 (RFA) | 0 | >35 | 0 |
| Tsivian et al. 2020 (77) | USA 1970-2012 | 561 | 68 | 68 | 35 (IQR 25-50) | 0 | 0 | 561 (319 RN, 242 PN) | 127 | 0 |
| Uzosike et al. 2018 (37) | USA 2009-2018 | 14 | NR | NR | NR | 14 | 0 | 0 | NR | 0 |
| Waldert et al. 2010 (78) | Austria 1994-2008 | 43 | 63 | NR | 37 (14-80) | 0 | 0 | 43 (16 RN, 27 PN) | NR | 0 |
| Wobker et al. 2016 (79) | USA 2000-2015 | 22 | 67 | 55 | 52 (10-120) | 0 | 0 | 21 (16 RN, 5 PN) | 30 (8-95) | 0 |
| Yen et al. 2009 (80) | Taiwan 1987-2002 | 16 | 54 | 50 | 66 (30-150) | 0 | 0 | 16 (16 RN) | 59 (12-189) | 0 |
| Yüksel et al. 2022 (81) | Turkey Dates NR | 55 | 64 | 69 | 44 (SD 18) | 0 | 0 | 55 (30 RN, 25 PN) | 76 | 0 |
| Zhang et al. 2015 (82) | China Dates NR | 4 | NR | NR | All $\leq$35 | 0 | 0 | 4 (RN) | 42 (6-67) | 0 |
| Zhang et al. 2009 (83) | USA 1989-2006 | 4 | 51 | 25 | 50 (30-85) | 0 | 0 | 4 (4 RN) | 31 (6-58) | 0 |
